# Circular Dichroism in the Second Harmonic Field Evidenced by Asymmetric Au Coated GaAs Nanowires

**DOI:** 10.3390/mi11020225

**Published:** 2020-02-23

**Authors:** Alessandro Belardini, Grigore Leahu, Emilija Petronijevic, Teemu Hakkarainen, Eero Koivusalo, Marcelo Rizzo Piton, Soile Talmila, Mircea Guina, Concita Sibilia

**Affiliations:** 1 SBAI Department, Sapienza University of Rome, 00161 Rome, Italy; grigore.leahu@uniroma1.it (G.L.); emilija.petronijevic@uniroma1.it (E.P.); concita.sibilia@uniroma1.it (C.S.); 2 Optoelectronics Research Centre, Tampere University, 33720 Tampere, Finland; teemu.hakkarainen@tuni.fi (T.H.); eero.koivusalo@tuni.fi (E.K.); marcelo.rizzopiton@tut.fi (M.R.P.); Soile.Talmila@tuni.fi (S.T.); mircea.guina@tuni.fi (M.G.)

**Keywords:** extrinsic chirality, second harmonic generation, GaAs nanowires, plasmonic coating

## Abstract

Optical circular dichroism (CD) is an important phenomenon in nanophotonics, that addresses top level applications such as circular polarized photon generation in optics, enantiomeric recognition in biophotonics and so on. Chiral nanostructures can lead to high CD, but the fabrication process usually requires a large effort, and extrinsic chiral samples can be produced by simpler techniques. Glancing angle deposition of gold on GaAs nanowires can (NWs) induces a symmetry breaking that leads to an optical CD response that mimics chiral behavior. The GaAs NWs have been fabricated by a self-catalyzed, bottom-up approach, leading to large surfaces and high-quality samples at a relatively low cost. Here, we investigate the second harmonic generation circular dichroism (SHG-CD) signal on GaAs nanowires partially covered with Au. SHG is a nonlinear process of even order, and thus extremely sensitive to symmetry breaking. Therefore, the visibility of the signal is very high when the fabricated samples present resonances at first and second harmonic frequencies (i.e., 800 and 400 nm, in our case).

## 1. Introduction

Three to five compounds have been utilized efficiently in photonic applications [1] as a result of their direct band gaps. Among these, the GaN semiconductor is important for its high transparency and good nonlinear properties in the visible range thanks to its high energy gap (3.4 eV) [2]. GaAs, for which the bandgap (1.42 eV) lies in the infrared, only recently have been utilized in the visible range. Indeed, a new category of applications has exploited their very high refractive index (around 4 in the visible range) to guide light in an effective way in nanostructures like nanowires (NWs) by using leaky waves [3,4], leading to different applications as emitters or even as laser sources [5]. 

By breaking the symmetry of the nanostructure–light interaction, it is possible to observe a circular dichroism (CD) due to the so-called extrinsic chirality or pseudo chirality [6,7,8]. Chirality is the lack of mirror symmetry [9], and can be probed using photoacoustic techniques that are sensitive to the differential absorptions of opposite-handed light [10,11,12] or by techniques sensitive to symmetry breaking such as second harmonic generation–circular dichroism (SHG-CD) [13,14]. In the case of extrinsic chirality, the high sensitivity of SHG is related to the fact that SHG can only occur in systems with broken inversion symmetry, enabling background-free measurements and leading to higher CD responses with respect other measurement systems [15]. The chiroptical responses of nanostructures have recently generated interest because most biomolecules are chiral, and their enantiomeric discrimination is relevant to industries such as pharmacology, agrochemicals and biotechnology, as well as for circularly polarized light emissions for communication and quantum optics applications [16,17]. Moreover, organic chiral molecules are used in field-effect transistor devices to detect or enhance the detection of circularly polarized light [18,19], while chiral oligothiophene thin films have shown interesting chiroptical properties that are useful to optoelectronic devices for imaging [20,21]. Thus, there is an evident need for a deep study of chiroptical effects at a nanoscale. 

Recently, we observed that GaAs nanowires (NWs) offered interesting waveguiding properties even for energies above the bandgap, thanks to the high refractive index of GaAs (in particular at 800 and 400 nm) [3]. We further verified, using photoacoustic spectroscopy, that when such GaAs NWs were partially covered in gold they exhibited strong extrinsic chirality due to the breaking of the symmetry induced by the asymmetric metal coating [11,12]. We also numerically investigated near-field chiral effects in high-refractive-index nanowires with [22] and without [23] an asymmetric plasmonic layer.

Here, we present SHG-CD measurements of gold coated GaAs NWs, confirming the strong presence of extrinsic chirality and leading to potential applications in chiral light emissions and manipulation.

## 2. Materials and Methods 

**Figure 1:**
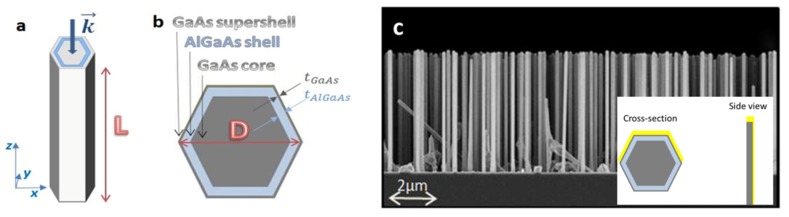
(**a**) Scheme of the NWs from [3]; (**b**) cross-section from [3]; (**c**) SEM image of Sample A (side view). The inset shows the scheme of the gold coating layer.

The structures under examination are nanowires of GaAs with a hexagonal cross section. They have a core of GaAs surrounded by a thin shell of AlGaAs to passivate the GaAs surface, around which there is a thin supershell of GaAs in order to prevent the oxidation of Al, as described in the scheme in Figure 1a. The geometric parameters of the four samples that were fabricated are depicted in Table 1 (the NW length *L*, the overall diameter *D*, AlGaAs shell thickness *t*_AlGaAs_, and GaAs supershell thickness *t*_GaAs_). 

The NWs were grown using molecular beam epitaxy on p-Si(111) wafers with lithography-free Si/SiO*x* patterns for defining the nucleation sites, as described in [24]. The lengths of the wires were about 5 microns while the diameters were in the 140–200 nm range (details in Table 1).

**Figure 2:**
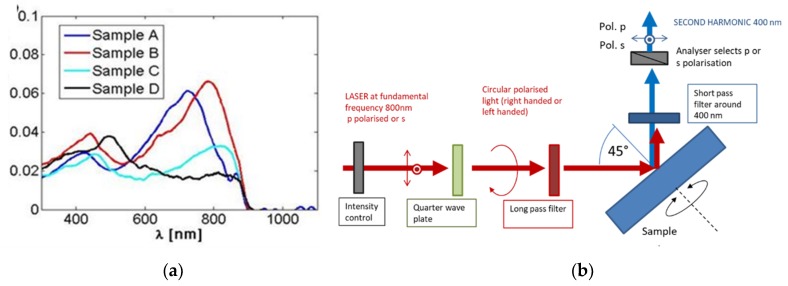
(**a**) Absorption spectra of the four samples without Au. Figure from [3]; (**b**) scheme of the second harmonic generation setup.

The as-fabricated NWs (before the gold coating) presented clear resonant modes in absorption [3] at 800 nm (close to the band edge), with the exception of Sample D (Figure 2a), and a second-order resonance at 400 nm, thus matching the first and second harmonic frequency of a standard Ti:–sapphire laser. This is due to the evidently larger overall diameter that red-shifts the modes into the transparent region in Sample D.

Half of each sample was asymmetrically coated with gold by glancing evaporation, as explained in more detail in [10,24]. The average Au thickness deposited on the sidewalls was around 15 nm, and the glanced evaporation resulted in the Au presence only on three out of six sidewalls, as described in the inset of Figure 1c. The absorption spectra of the samples coated with gold were similar to the ones without gold, except for a slight broadening of the resonant features, as shown in [10]. The Au-free NWs were used as reference samples for the optical measurements. 

The samples were then measured by a SHG-CD setup shown in Figure 2b.

At 800 nm, a linearly polarized Ti–sapphire fs laser with a pulse duration of 100 fs and a repetition rate of 80 MHz was used on the sample at an incidence angle of 45°. The average power was attenuated with a chopper below 1 GW/cm^2^ to avoid sample damage or multiphoton processes. A quarter waveplate was used to obtain either left circularly (LCP) or right circularly (RCP) polarized light. A long-pass filter removed any spurious signals at the second harmonic wavelength (400 nm). 

The sample itself was mounted on an automatic azimuthal rotation stage whose axis was aligned with the incidence point. The SHG signal produced in the reflection was then detected after passing through a short pass filter that removed the first harmonic pump. The SHG was analyzed in the s (vertical) or p (horizontal) polarization state by an analyzer (linear polarizer). Since the output SHG signal in p state was larger than the one in s state, we report explicitly only the p signal in this manuscript. The signal was further filtered by a narrow bandpass filter centered at 400 nm (FWHM 10 nm) and finally detected by a photomultiplier tube in a gated photon counting regime.

As blank references, we also measured the bare p-Si(111) wafer (thickness of around 400 microns) and a sonicated sample where the wires were removed, leading to a flat GaAs layer of about 50 nm on p-Si(111) substrate. 

All the SHG measurements were performed with the same intensity level of the laser.

## 3. Results

**Figure 3:**
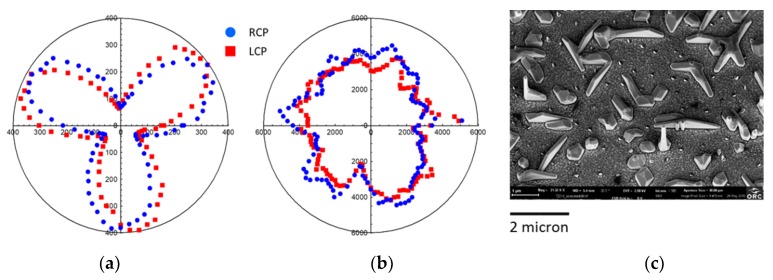
(**a**) p-polarized SHG signal from the bare p-Si(111) sample for RCP and LCP light with a maximum signal of 400 counts; (**b**) p-polarized SHG signal from the flat GaAs sonicated sample for RCP and LCP light with a maximum signal of 5500 counts; (**c**) SEM image of the sonicated GaAs sample. Horizontal residual GaAs crystallites are evident. In the measurements the azimuthal rotation angle is relative.

In Figure 3a, we show the measured p-polarized SHG signal from the blank reference sample of the bare p-Si(111) as a function of azimuthal rotation of the sample for two orthogonal circular polarization states of the laser pump (RCP and LCP), while in Figure 3b we show the measured p-polarized SHG signal of the flat GaAs sonicated substrate under the same experimental conditions.

The Si substrate response showed a clear, but low, SHG signal with three-fold symmetry, as expected from the 111 crystallographic orientation. Si is a third order nonlinear material, and thus the SHG signal is due to surface contribution. There was a small CD due to a normal incidence on the asymmetric 111 surface.

Meanwhile, the flat GaAs sample showed a larger SHG signal (×14 times the one of Si) due to its bulk nonlinear coefficient [25]. In Figure 3c, the SEM image of the reference flat GaAs sample is shown, which was obtained by Sample C after sonication in order to remove the NWs. In the figure, the largest objects are the parasitic crystallites. The orientation of these crystallites correlated with the silicon substrate, and their microstructures showed a three-fold geometrical symmetry with two possible orientations for the crystallites, one being rotated by 180 degrees with respect to the other, leading to a six-fold microstructure symmetry (also evidenced by the hexagonal shaper of the SHG measurements). The roughness on the Si surface of the substrate in Figure 3c is parasitic polycrystalline AlGaAs/GaAs, which formed during the shell growth. Since this layer grew on the oxide-covered areas of the Si substrate, the orientation of these small crystallites was random and gave rise to the main isotropic SHG signal in Figure 3b.

**Figure 4:**
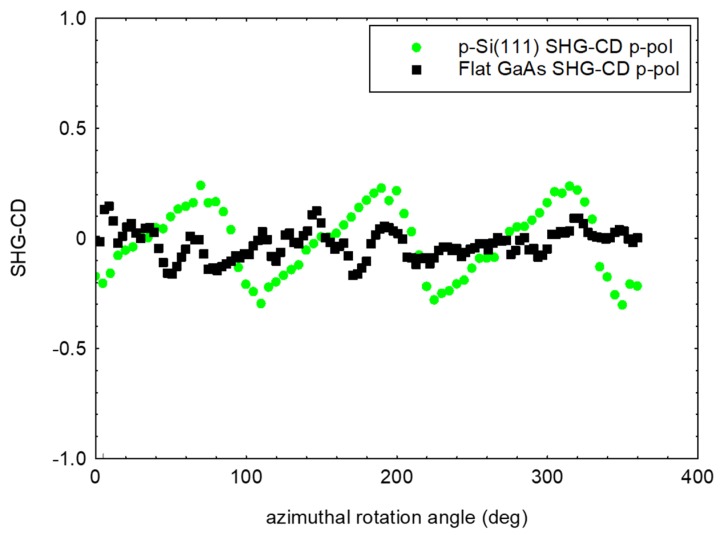
SHG-CD signal of the p-Si(111) reference and of the flat GaAs substrate. In the measurements the azimuthal rotation angle is relative.

In Figure 4, we show the measured SHG-CD in p-pol light for both Si(111) and the sonicated GaAs samples, defined as

(1) $$\text{SHG-CD}=\frac{I_{LCP}^{\left(2\omega \right)\;}-I_{RCP}^{\left(2\omega \right)\;}}{I_{LCP}^{\left(2\omega \right)\;}+I_{RCP}^{\left(2\omega \right)}}$$

where $I_{LCP}^{\left(2\omega \right)}$is the intensity of SHG signal when the fundamental pumping light is circularly left polarized, and $I_{RCP}^{\left(2\omega \right)}$is the intensity of SHG signal when the fundamental pumping light is circularly right polarized.

In the case of Si(111), the SHG-CD was regular even when it was lower than 0.2, while in the case of GaAs it was randomly distributed at values lower than 0.1.

**Figure 5:**
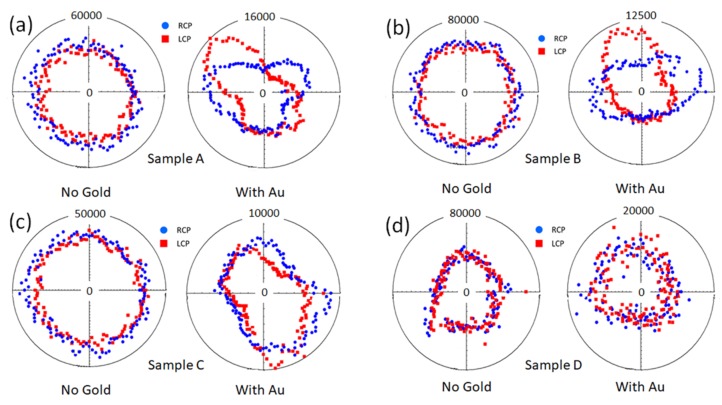
p-polarized SHG signal from: (**a**) Sample A; (**b**) Sample B; (**c**) Sample C; (**d**) Sample D. On the left side of each panel the samples without Au coating for RCP and LCP light are shown, while on the right side of each panel, the samples with Au coating are shown. Adapted from [26]. In the measurements the azimuthal rotation angle is relative.

On the left side of each panel of Figure 5, the measured p-polarized SHG signal from Samples A,B,C,D are shown without gold as a function of the azimuthal rotation of each sample for two orthogonal circular polarization states of the laser pump (RCP and LCP), while on the right side of each panel of Figure 5, the measured p-polarized SHG signal of Samples A,B,C,D are shown with asymmetric gold coating. It is evident that the asymmetry in the structures of Samples A,B,C (with Au) led to a strong difference in the SHG signal as a function of the handedness of the circular polarized light, while the SHG response of Sample D with Au was very similar to its uncoated counterpart. 

By concerning the magnitude of the SHG signal, the maximum signal was 50,000 counts for Sample A without Au, while the Au coating decreased the SHG signal to 16,000 counts. The magnitude of SHG for sample A (no Au) was nine times larger than the magnitude of the flat GaAs sample, while the magnitude of Sample A (Au) was three times larger. This indicates that the SHG was enhanced by the geometrical resonances of the GaAs NWs, and that the Au layer did not increase the SHG signal but hindered it by selective absorption of one handedness of circular polarized light respect to the other, leading to a lower signal, but with a higher CD.

Samples B,C,D without Au showed maximum signals of 60,000, 40,000, and 40,000 counts, respectively, which decreased to 10,000, 10,000, and 14,000, counts, respectively, when coated with Au [26]. 

**Figure 6:**
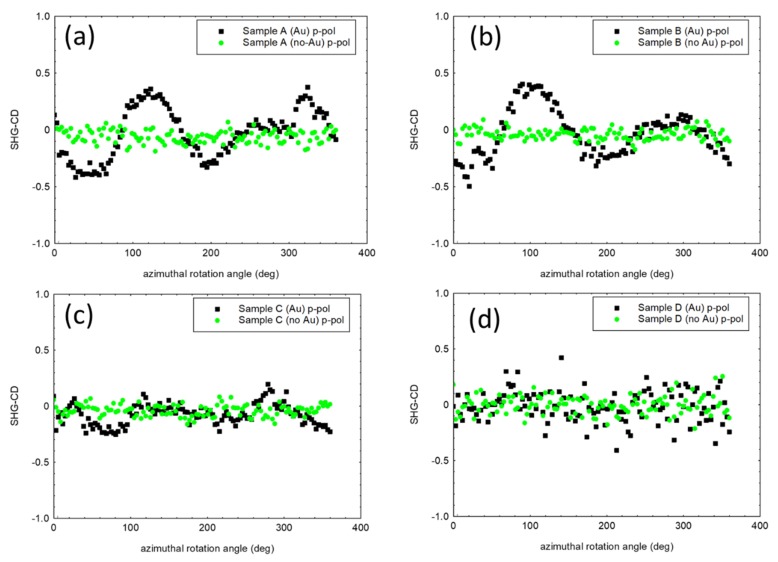
SHG-CD signal of the Samples without Au and with Au. SHC-CD for: (**a**) Sample A; (**b**) Sample B; (**c**) Sample C; (**d**) Sample D. In the measurements the azimuthal rotation angle is relative.

In Figure 6, we show the SHG-CD of the Samples calculated by Equation (1).

For all the four samples, the symmetric samples provided negligible SHG-CD, despite the large magnitude of SHG signals. Sample A with Au, showed a SHG-CD as high as 0.5. Similarly, the asymmetric Sample B with Au led to a SHG-CD of 0.5.

By considering Sample C, despite a SHG magnitude comparable with previous cases, the SHG-CD was dramatically decreased at a level of about 0.3-0.25 due to the resonant behavior of Sample C around 800 nm (see Figure 2a). This is because the diameter of the wires achieved larger values and thus red-shifted the spectral position of the resonance [3,10]. 

In the case of Sample D, the SHG-CD was negligible for both symmetric and asymmetric samples due to the complete lack of resonance at 800 nm (see Figure 2a) [3]. Here, the diameter of the wires was so large that the modes fell in the transparent region of the GaAs spectrum at larger wavelengths with respect to the band gap.

## 4. Discussion 

Even though the lithography-free and self-assembled growth methods used for wire growth suffered from an intrinsic degree of disorder, we nevertheless saw a good agreement in the general trend of the SHG-CD signal as a function of the wires’ diameters, and we were able to quantitatively compare different samples with a reasonable degree of approximation. In these experiments, we demonstrated two main issues. The first one is the possibility for strong circular dichroic responses from asymmetric samples formed by GaAs NWs partially covered in a thin Au film. This could have applications in different fields, including the ability to generate photons in a SH field, while selective pumping with circular polarized light could be viewed as a possibility for boosting the processes of circular polarized photon generation or absorption. Secondly, we observed that geometric resonance is an essential feature in this extrinsic chiral behavior. Only when resonant leaky modes were present was the geometric-induced CD enhanced in the second harmonic field. The resonance can be finely tuned by changing the diameter of the NWs. We passed from 138-nm diameter wires that showed a strong resonance around 750 nm, to 151-nm diameter wires with a strong resonance at 810 nm. As the diameter increased to 165 nm, the resonance shifted to longer a wavelength (850 nm), decreasing its magnitude as the wavelength approached the transparent region of GaAs. In these cases, we passed from a large CD of 50% (0.5) to a CD of 25%. Finally, for larger-diameter wires (197 nm), the resonance completely disappeared in the GaAs bandgap region, and negligible CD was present, despite the strong SHG signal, destroying any information about the geometrically induced asymmetry of the sample.

## Figures and Tables

**Table 1:** Fabrication parameters of the nanowire (NW) samples. Data from [3].

Sample	*L* (nm)	*D* (nm)	*t*_AlGaAs_ (nm)	*t*_GaAs_ (nm)
A	4750 ± 34	138 ± 5	3.5	0.7
B	5190 ± 64	151 ± 5	8.6	1.7
C	4600 ± 52	165 ± 6	11.7	5.8
D	4690 ± 47	197 ± 9	27.7	5.5
